# The Usability of a Touchpad Active Video Game Controller for Individuals With Impaired Mobility: Observational Study

**DOI:** 10.2196/41993

**Published:** 2023-08-03

**Authors:** Christen J Mendonca, Laurie A Malone, Sangeetha Mohanraj, Mohanraj Thirumalai

**Affiliations:** 1 School of Health Professions The University of Alabama at Birmingham Birmingham, AL United States

**Keywords:** active video games, exergames, usability, enjoyment, disability, mobility limitation, mobility impairment

## Abstract

**Background:**

Video games are a popular sedentary activity among people with impaired mobility; however, active video game hardware typically lacks accessibility and customization options for individuals with mobility impairments. A touchpad video game system can elicit moderate physical activity in healthy adults; however, it is unclear if this system is usable by adults with impaired mobility.

**Objective:**

The purpose of this study was to assess the usability of a touchpad video game controller system adapted for adults with impaired mobility. Additional outcomes explored were enjoyment, perceived exertion, self-efficacy, participant feedback, and researcher observations of gameplay.

**Methods:**

Participants played several video game titles for 20 minutes with a touchpad video game controller as they stood or sat in a chair or their wheelchair. Usability was assessed with the System Usability Scale (SUS) and the Health Information Technology Usability Evaluation Scale (Health-ITUES) surveys after gameplay. After each video game, participants reported enjoyment using a visual analog scale (0 to 100 mm) and a rating of perceived exertion using the OMNI 0 to 10 scale. Self-efficacy was measured before and after gameplay. Participants provided feedback at the end of their session.

**Results:**

In total, 21 adults (6 females and 15 males) with a mean age of 48.8 (SD 13.8) years with various mobility impairments participated in this study. The touchpads received mean usability scores on the SUS 80.1 (SD 18.5) and Health-ITUES 4.23 (SD 0.67).

**Conclusions:**

The SUS scores reported suggest the touchpad system is “usable”; however, the Health-ITUES scores were slightly below a suggested benchmark. Participants reported moderate to high enjoyment but perceived the exertion as “somewhat easy.” Self-efficacy was moderate to high and did not differ pre- to postgame play. The participants regarded the touchpads as novel, fun, and entertaining. The generalizability of our results is limited due to the heterogenous sample; however, our participants identified several areas of improvement for future iteration.

## Introduction

Habitual physical activity improves health and quality of life; however, half of the people with a disability in the United States are categorized as physically inactive [1-4]. While not every person with a disability possesses a mobility impairment, individuals with impaired mobility encounter personal and environmental barriers that affect participation in physical activity (eg, lack of transportation, poor facility access, and inexperienced staff) [5-8]. While physical activity research typically focuses on traditional exercise, and sport programs [1,2], home-based inclusive alternatives such as an arm ergometer may be viewed as tedious and boring [9]. Additional opportunities to engage in healthy physical activity are needed for individuals with mobility impairments. Technology, such as video games, can augment traditional exercise and may increase adherence to a healthy lifestyle [10].

While half of adults in the United States engage in sedentary video game play [11], active video games (AVGs) have been identified as a means to promote leisure time physical activity in adults and children [12-14]. AVGs typically integrate active trunk and limb movements to control onscreen video game actions (eg, Nintendo Wii and Xbox Kinect). Research indicates that increased energy expenditure is elicited in persons with impaired mobility during AVG play and may mitigate the effects of sedentary behavior [15-20] Additionally, AVGs can circumvent barriers to physical activity such as transportation and facility access among individuals with impaired mobility [8]. However, most current AVGs are not typically inclusive of those who have difficulty standing, weakness in their lower extremities, poor motor control, or use an assistive device [21,22].

Given that AVGs can foster feelings of autonomy, competency, and relatedness, these games may fulfill basic psychological needs and augment the enjoyment derived from participation in physical activity [23,24]. Furthermore, AVGs have been shown to be an enjoyable opportunity to increase weekly physical activity minutes [19]. Additionally, enjoyment exhibits a stronger influence on positive exercise behavior compared to health or fitness motives [25]. Because people are more likely to participate in physical activity if they are certain they can do it [26], self-efficacy has been found to highly correlate with positive physical activity behavior [27]. Increased self-efficacy is related to increased AVG enjoyment [28,29], exercise adherence [30], exercise duration [31], and AVG approval [32].

The research and development team with the Rehabilitation Engineering Research Center on Interactive Exercise Technologies and Exercise Physiology for People with Disabilities previously showed that AVG play can be adapted for wheelchair users [33], be enjoyable, and elicit light to moderate physical activity [18]. A newly developed device called the GAIMplank was demonstrated to be usable and accessible among individuals with impaired mobility [13]. Another video game controller called the touchpad system (TPS) was originally designed as an easy to assemble low-fidelity proof of concept to elicit physical activity using sedentary video games. Research demonstrated that sedentary video games could be adapted to elicit moderate physical activity in healthy adults by using the TPS [34]. However, the TPS system has not been tested among adults with impaired mobility. The aim of this study was to assess usability of the TPS among individuals with impaired mobility and examine enjoyment, perceived exertion, task self-efficacy, and player feedback regarding use of the system.

## Methods

### TPS Development

The TPS is an internally developed AVG controller designed to add physical activity to sedentary video games. This prototype translates physical contact into video game commands. Because the TPS is recognized as a USB controller, this system can provide commands to any video game title that features controller support. The current proof-of-concept has been adapted for use by people with impaired mobility.

We constructed 6 touchpads using a particle board base, conductive aluminum tape, electrical wire, and duct tape. The touchpads were wired to a MAKEY-MAKEY circuit board (MAKEY-MAKEY LLC, Santa Cruz, CA) that was connected to the video game computer as a controller. All but 1 touchpad measured 20 cm × 20 cm, and the final touchpad was larger (61 cm × 31 cm). Each touchpad was placed on an adjustable stand that enabled varying height, distance, and orientation (vertical to horizontal). The smaller touchpads were placed in front or to the side of the player. The larger touchpad was placed behind the player.

To ensure safety, adjustable parallel bars were located on both sides of the player during gameplay. The participants played video games approximately 1.8 m away from a flat-screen television. Touchpads were placed so they did not block the player’s view of the screen. All wires were secured away from the player to prevent any trip hazard during gameplay.

### Design and Setting

This was a cross-sectional usability testing study. We assessed usability and embedded participant feedback to help explain our results. All written consent and data collection took place at the University of Alabama at Birmingham within the RERC RecTech Exercise Science and Technology Laboratory. Participants attended a single 60- to 90-minute session to test the usability of the system.

### Ethics Approval

The procedures of this study were approved by the University of Alabama at Birmingham’s institutional review board (IRB 300003265).

### Participant Recruitment

We recruited participants from the local community of Birmingham, AL, using flyers and word of mouth. Sample size estimates were based on identifying common usability barriers for the system, and issues specific to the 3 modes of play (standing, chair sitting, and wheelchair sitting). According to Cazañas et al [35], a sample of 17 individuals would reasonably identify 80% of common problems in the system, and groups of 4 to 9 are sufficient to identify problems specific to the mode of play. In total, 21 participants were recruited to account for modest attrition.

Interested individuals were included if they were an adult 18 to 75 years of age, had a self-reported mobility impairment, and possessed the ability to exercise with their upper extremities. Individuals were excluded if they were unable to converse in English, weighed greater than 181.4 kg (400 lbs), had significant visual impairment that prevented them from seeing a large flat-screen television, had cardiovascular disease within the previous 6 months, had severe pulmonary disease or renal failure, currently pregnant, ongoing exacerbation of a health condition, or any other condition that would interfere with testing procedures. Participants received a US $50 gift card at the end of their visit.

### Measures

Participant usability of the TPS was assessed using the System Usability Scale (SUS) and the Health-Information Technology Usability Evaluation Scale (Health-ITUES). The SUS is comprised of 10 statements, and participants rate their agreement of each statement with a 5-point Likert scale from “strongly disagree” to “strongly agree.” Across 206 usability tests and 2324 responses, the SUS was found to be a robust and reliable (α=.91) tool to measure usability [36,37]. The SUS is robust to small sample sizes and applicable to many systems [36,38,39]. The SUS can produce a score from 0 to 100 and a score equal to or greater than 68 indicates above-average usability [37,39]. In addition to the single score, factor analyses suggest a 2-factor structure of learnability and usability [40]. The SUS scores were summed and converted from a 0 to 40 into a 0 to 100 scale, and a score of 68 was the threshold to indicate the TPS is “usable” [36].

Similar to the SUS, the Health-ITUES is made up of 20 statements that participants rate on a 5-point Likert scale from “strongly disagree” to “strongly agree” [41]. The subscales of the Health-ITUES demonstrate high internal consistency and reliability (α=.85-.92) [42], and the construct validity of the Health-ITUES has been established [43]. Health-ITUES scores can typically be difficult to generalize; however, a cut-point score of 4.32 has been suggested as a cutoff to represent a system is “usable” [44]. Health-ITUES was scored by calculating a mean score for each subscale, and a total score by calculating the mean of the subscale scores.

Participants reported their enjoyment with a visual analog scale (VAS), which is a 10-cm scale with anchor phrases at each end [45]. The anchors for our enjoyment VAS were from “not enjoyable at all” to “most enjoyable.” Using an electronic tablet computer, participants touched the line on the spot that best represented their enjoyment. The length of the line is used as a measure of their enjoyment and is reported to be the closest 0.1 cm. Enjoyment VAS scores were converted from centimeters to millimeters and reported from 0 to 100.

Participants rated their perceived exertion using the OMNI 10-point ratings of perceived exertion (RPE) scale [46]. The scale was shown and explained before data collection. Participants could point to or say a number from 0 (extremely easy) to 10 (extremely hard). A researcher confirmed the RPE number with the participant before recording the response. A score of 4-6 would be considered somewhat easy to somewhat hard.

Task self-efficacy was assessed using the video game play appraisal (Multimedia Appendix 1). This scale asks respondents to rate their certainty on 6 dimensions of video game play with the TPS from 0 (no certainty) to 10 (absolute certainty). The scale was created based on expert recommendations [26,47], and the video game play dimensions were chosen to represent the key steps in video game interaction [48]. Among a sample of 30 healthy adults, this scale exhibited high internal consistency (α=.95), and good test-retest reliability with an ICC_3,2_ (intraclass correlation coefficient) 0.83 (95% CI 0.62-0.91) [34]. The participants’ understanding of the task itself is vital to the validity of a task self-efficacy scale [47]; therefore, prior to video game play, each participant watched an instructional video illustrating how the TPS is used while sitting in a fixed chair, standing, and seated in a wheelchair.

### Participant Feedback and Researcher Observations

After the participant completed usability surveys, they provided open-ended feedback through surveys and semistructured interviews. The survey and interview questions were designed to explore the participant’s perspectives on accessibility, overall experience, and identify areas of improvement. The initial 8 participants were asked to answer open-ended questions on their own. To gain richer feedback from the responses, the remaining participants were interviewed by a member of the research staff using an interview guide (Multimedia Appendix 2). Interviews were audio recorded and later transcribed. The transcriptions were merged with responses to the open-ended questions where appropriate. Researchers documented written observations from gameplay sessions regarding modifications, adaptations, areas of improvement, and suggestions for future touchpad iteration.

### Instruments

The TPS is an alternative video game controller that substitutes typical game controls with large movements. The system is designed to control a wide variety of video games available on PC and is easily adapted to users of varying abilities.

The TPS consists of 6 individual touchpads, circuit board, and laptop computer. Each touchpad is a square of particle board with a surface of conductive tape that was placed within a flexible stand that could be adjusted in any direction. All touchpads were wired into a small circuit board microcontroller (Makey-Makey LLC, Santa Cruz, CA) that converts electrical input into computer keys. Touchpads were connected to computer keys that corresponded with video game controls. The microcontroller functions as a video game controller and was connected to the PC via USB cable. The PC was used to run the 4 video game titles that participants played (Table 1). A 127-cm television flat screen was used to display the games 1.8 m in front of the participant.

**Table 1:** Description of video game titles and commands required for gameplay.

Game	Genre	Game description	Commands required
Flower	Flight; exploration	Player moves a floating flower petal around a peaceful meadow	Left, right, forward (up), backward (down)
PAC-MAN Championship Edition DX+	Arcade; action	Navigate a maze to collect points by eating pellets and avoid ghosts that chase the player	Left, right, forward (up), backward (down), bomb
Super Destronaut	Shooter	Player moves a spaceship left and right, shoots aliens, and dodges attacks	Left, right, shoot
Super Indie Karts	Racing	Player steers a go-kart around a track against computer opponents and uses power-ups	Steering left and right, activate special items

**Figure 1:**
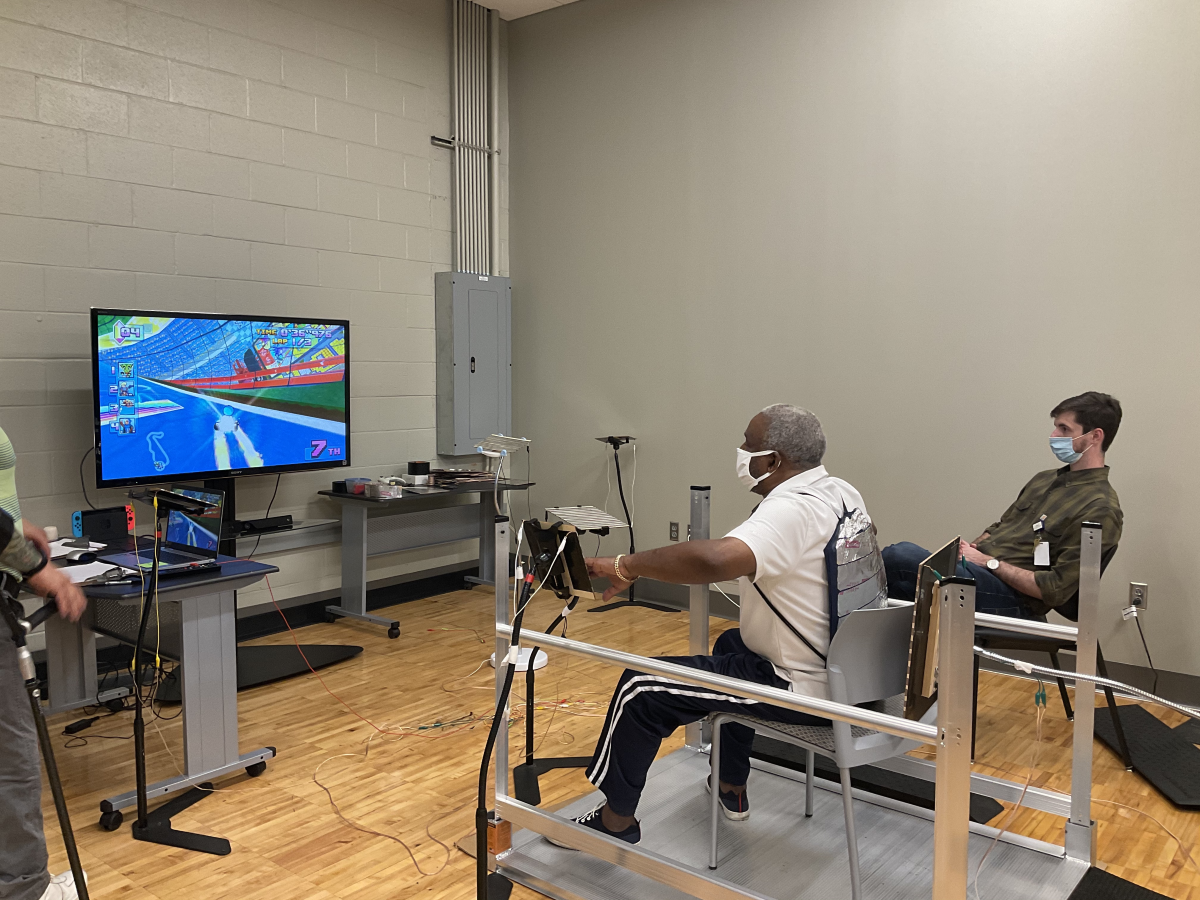
Participant using the touchpad system sitting in a chair.

**Figure 2:**
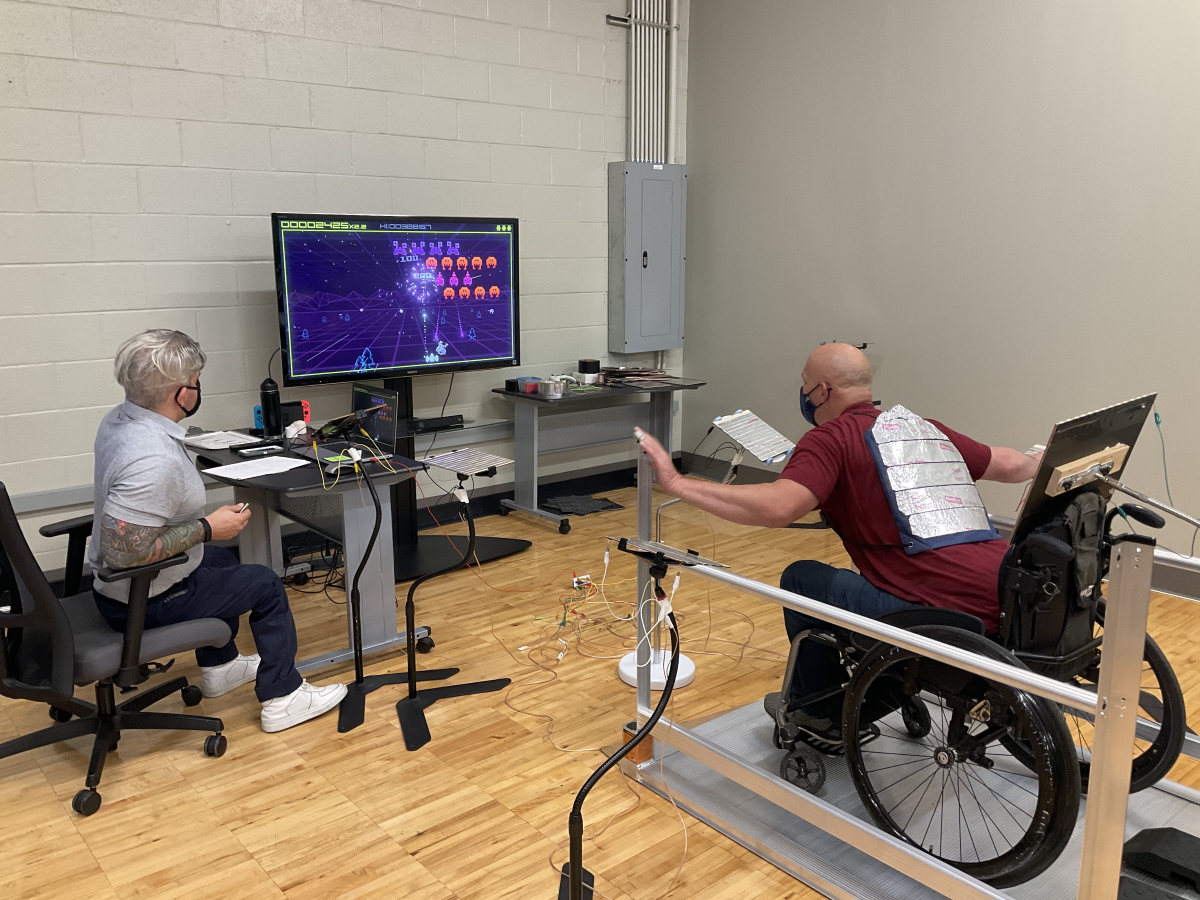
Participant using the touchpad system sitting in their wheelchair.

**Figure 3:**
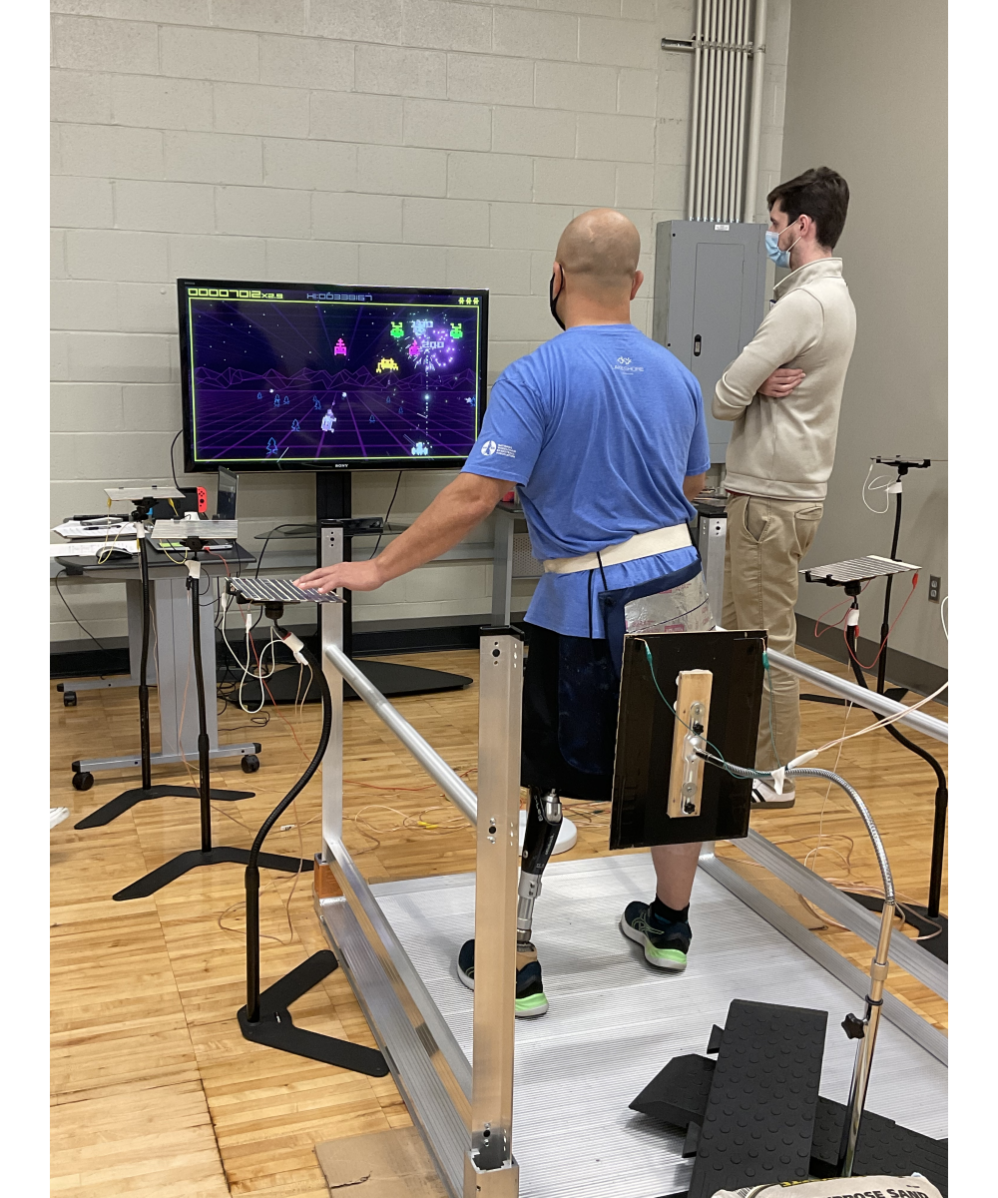
Participant using the touchpad system standing.

To use the TPS, the player sat in a chair (Figure 1), sat in their wheelchair (Figure 2), or stood (Figure 3) within a set of parallel bars that they could hold or lean onto for added stability. Once the player was situated, the touchpads were moved around them at a distance that was within reach but made the player lean and reach to make physical contact. The hand or forearm activated touchpads in the front and to the sides of the player were 20 cm × 20 cm in size and placed at trunk height to the players. A larger 61 cm × 31 cm touchpad was created to go behind the player and was placed at scapula height for the seated player and hip height for standing players. To activate this larger touchpad, the participant wore a light harness with a conductive material either around their shoulders (seated) or hips (standing). The player leaned rearward to make contact and activate the larger touchpad.

### Procedures

Each person was screened for eligibility by phone before visiting the laboratory. The visit began with reviewing test procedures and then obtaining written informed consent from the participant. Participants answered a baseline questionnaire that asked about their demographics, prior video game experience, and physical activity habits. Next, the participant’s heart rate and blood pressure were measured to ensure the participant was safe to engage in physical activity.

Before the TPS was used, the participant was shown the play area and watched an instructional video that demonstrated both the seated and standing use of the system. After watching the video, the participant rated their self-efficacy before playing. The TPS session consisted of playing 4 different video game titles for 5 minutes each with 5 minutes of rest in between each video game title. The TPS session began with playing a relaxing slow-moving game (Flower) to enable the participant to acclimate to the necessary movements. The sequence of the remaining 3 video game titles was randomized. Immediately at the end of playing each video game, participants were asked to provide an RPE and enjoyment score. At the end of the TPS session, the participant rated their self-efficacy and completed the SUS and Health-ITUES. The entire session was recorded with the participant’s consent.

### Data Analyses

Participants’ characteristics are reported as mean (SD) and range. The interitem reliability of SUS, Health-ITUES, and self-efficacy scores was assessed using Cronbach α. A 2-way mixed effects ICC_3,2_ was used to assess test-retest reliability of self-efficacy scores. Because self-efficacy and baseline questionnaire responses were not normally distributed, nonparametric statistics were used. A Wilcoxon signed rank test was used to determine if self-efficacy differed from pre- to postgame play. Spearman ρ correlations were calculated to explore relationships among physical activity minutes, video game minutes, usability, enjoyment, perceived exertion, and self-efficacy. For consistency, nonnormally distributed data are reposted as mean (SD). Interview responses and open-ended questions were reviewed by a member of the research team to extract common themes and feedback. Data were analyzed using SPSS (version 27; IBM Corp).

The audio recordings of the participant feedback and written researcher observations were transcribed and combined into the same database. Participant feedback and researcher observations were examined and organized by 2 members of the research team separately. The same 2 researchers classified these data into 3 main categories: accessibility, overall experience, and areas of improvement.

## Results

### Participant Characteristics

Usability testing was completed by 21 participants 48.8 (13.8) years of age. Our sample consisted of 15 males and 6 females and reported their race as either Black (n=8), White (n=12), or Asian (n=1). Participants reported impaired mobility due to stroke (n=9), spinal cord injury (n=3), amputation (n=3), cerebral palsy (n=2), spina bifida (n=2), or other (n=2). The primary mode of mobility included walking without assistive device (n=7), cane (n=6), prosthetic leg (n=1), rollator walker (n=1), and manual wheelchair (n=6). Participants used the TPS to play either standing (n=8), seated in a 4-legged chair (n=7), or seated in their own manual wheelchair (n=6). All participants were able to complete data collection. Some participants required slight modifications to play such as altering the height of the touchpad, moving the touchpad closer to the player, and adjusting the tilt of the touchpad.

### Measures

Participant responses to the baseline questionnaire can be found in Table 2. When asked to rate enjoyment of certain activities from 1 “strongly disagree” to 5 “strongly agree,” participants reported high agreement with both leisure time physical activity 4.6 (0.6) and video games 4.1 (1.1). However, there was high variability in our sample with reported weekly physical activity of 375 (257) minutes and video game play of 398 (643) minutes. There were no sex differences found for any baseline or outcome measures. The participants preferred playing video games on either video game consoles or cell phones.

**Table 2:** Participant responses to baseline questionnaire questions.

Question	Mean (SD)
Enjoys leisure time physical activity, 1=strongly disagree, 5=strongly agree	4.6 (0.6)
Weekday leisure time physical activity minutes	259 (177)
Weekend leisure time physical activity minutes	116 (103)
Enjoys playing video games, 1=strongly disagree, 5=strongly agree	4.1 (1.1)
Weekday video game play minutes	264 (462)
Weekend video game play minutes	138 (219)

Usability and subscale scores are reported with summary group scores in Table 3. The SUS demonstrated good reliability (α=.89). Participants reported above-average usability with an average SUS scores of 80.1 (SD 18.5). The Health-ITUES demonstrated excellent reliability (α=.92). However, the mean Health-ITUES score of 4.23 (SD 0.67) did not meet the suggested cutoff of 4.32.

**Table 3:** Self-report touchpad system usability scores.

Mode of play	SUS^a^: overall		SUS: usability subscale			SUS: learning subscale			Health-ITUES^b^	
	Mean (SD)	Range	Mean (SD)	Range	Mean (SD)		Range	Mean (SD)		Range
All players	80.1 (18.5)	25-100	80.4 (18.0)	28-100	79.2 (27.5)		13-100	4.23 (0.67)		2.22-5.00
Chair sitting	68.6 (26.0)	25-95	79.1 (18.2)	28-97	79.2 (28.6)		13-88	3.89 (0.91)		2.22-4.80
Standing	87.2 (10.0)	72.5-100	79.3 (18.4)	72-100	86.0 (19.1)		75-100	4.51 (0.45)		3.75-5.00
Wheelchair	84.2 (11.7)	65-100	76.8 (24.0)	66-97	84.6 (23.9)		63-100	4.25 (0.48)		3.70-4.75

^a^SUS: System Usability Scale.

^b^Health-ITUES: Health Information Technology Usability Evaluation Scale.

Overall enjoyment and perceived exertion scores including scores by video game title can be found in Table 4. Across all video games, participants moderately enjoyed gameplay with an overall mean VAS score of 70 (SD 22) mm. Perceived exertion of the participants was approximately “somewhat easy” with a mean RPE of 4.3 (SD 2.0).

**Table 4:** Enjoyment and ratings of perceived exertion by game and mode of play.

Game		Enjoyment (0-100 mm)			Rating of perceived exertion (0-10)	
		Mean (SD)	Range	Mean (SD)		Range
**All players, across games**		70.2 (22.4)	12-94	4.3 (2.0)		0.8-7.8
	Chair sitting	67.1 (34.5)	12-94	5.3 (1.8)		2.5-7.8
	Standing	72.8 (16.1)	42-93	3.4 (2.4)		0.8-7.5
	Wheelchair	70.4 (13.9)	50-88	4.2 (1.1)		3.0-5.8
**Flower**		62.4 (25.8)	4-97	2.7 (2.0)		0-6
	Chair sitting	62.6 (37.1)	4-97	3.6 (2.0)		1-6
	Standing	61.3 (11.6)	49-79	2.3 (2.0)		0-6
	Wheelchair	63.8 (28.6)	11-90	2.3 (2.1)		0-6
**PAC-MAN Championship Edition DX+**		76.8 (29.2)	0-99	5.0 (2.4)		1-10
	Chair sitting	70.1 (36.6)	0-99	6.1 (1.8)		3-9
	Standing	74.0 (33.0)	0-97	4.0 (3.0)		1-10
	Wheelchair	88.3 (6.3)	80-99	5.0 (1.5)		3-7
**Super Destronaut**		79.6 (21.9)	17-100	4.6 (2.6)		0-10
	Chair sitting	74.4 (31.0)	17-99	6.4 (2.2)		4-10
	Standing	85.1 (14.2)	64-100	5.0 (2.2)		0-6
	Wheelchair	78.2 (19.7)	47-98	3.0 (1.9)		1-7
**Super Indie Karts**		62.0 (31.4)	0-97	4.8 (2.6)		0-9
	Chair sitting	61.4 (40.3)	0-94	5.0 (3.3)		1-9
	Standing	70.6 (26.3)	10-97	4.3 (2.7)		0-8
	Wheelchair	51.2 (27.8)	0-83	5.2 (1.6)		4-8

Self-efficacy scores by dimension and mode of play are included in Table 5. The 6 dimensions of the self-efficacy scale exhibited excellent internal consistency for both the preplay (α=.95) and postplay measures (α=.97). Similarly, the self-efficacy scale demonstrated good test-retest reliability with average measures (ICC_3,2_=0.88, 95% CI 0.71-0.95; *P*<.001). Participants were highly certain in their abilities to use the TPS (mean 7.6, SD 2.2). Self-efficacy did not differ from pre- to postplay.

**Table 5:** Self-efficacy scores by dimension and mode of play.

Video game appraisal question			All				Chair sitting				Standing				Wheelchair		
			Pre		Post		Pre		Post		Pre		Post		Pre		Post
**Maintaining focus throughout a 5-minute session**																	
	Mean (SD)	8.9 (2.0)		8.5 (2.4)		7.0 (2.6)		6.6 (2.8)		9.8 (0.7)		9.1 (1.8)		10.0 (0.0)		10.0 (0.0)	
	Range	3-10		1-10		3-10		1-10		8-10		5-10		10-10		10-10	
**Seeing and hearing all the game information**																	
	Mean (SD)	8.5 (2.2)		8.5 (2.5)		7.0 (2.5)		6.9 (3.7)		9.4 (0.9)		9.4 (0.9)		9.0 (2.4)		9.3 (1.0)	
	Range	3-10		0-10		3-10		0-10		8-10		8-10		4-10		8-10	
**Reacting fast enough to choose a next action**																	
	Mean (SD)	6.7 (2.2)		6.8 (2.6)		5.7 (2.9)		4.9 (2.9)		7.4 (1.9)		7.8 (1.9)		6.8 (1.7)		7.8 (1.9)	
	Range	0-10		0-10		0-9		0-8		5-10		5-10		4-9		6-10	
**Determining strategies to move during play**																	
	Mean (SD)	7.2 (2.2)		7.1 (2.6)		5.6 (2.6)		5.1 (3.0)		8.1 (1.4)		8.5 (1.8)		7.8 (2.0)		7.7 (1.9)	
	Range	1-10		0-10		1-9		0-8		6-10		5-10		4-10		6-10	
**Coordinating body movements to carry out a strategy**																	
	Mean (SD)	7.1 (2.2)		7.0 (2.5)		5.9 (3.0)		4.9 (2.9)		8.3 (1.2)		7.8 (1.8)		7.2 (1.2)		8.3 (1.4)	
	Range	0-10		0-10		0-9		0-8		7-10		5-10		6-9		7-10	
**Moving well enough to maintain successful play**																	
	Mean (SD)	7.3 (2.5)		7.6 (2.8)		5.7 (3.1)		4.9 (3.1)		8.5 (1.2)		9.3 (1.2)		7.7 (2.1)		8.7 (1.4)	
	Range	0-10		1-10		0-9		1-8		7-10		7-10		5-10		7-10	
**Total**																	
	Mean (SD)	7.6 (2.0)		7.6 (2.4)		6.1 (2.6)		5.5 (2.9)		8.6 (1.0)		8.6 (1.2)		8.1 (1.3)		8.6 (1.1)	
	Range	1-10		0-10		1-9		0-9		8-10		7-10		6-10		8-10	

We found no relationships between weekly physical activity or video game play minutes and outcome variables. Both usability measures were moderately correlated with each other (*r_s_*=0.50). Enjoyment and perceived exertion did not exhibit a relationship with other outcome variables. Self-efficacy after using the TPS was moderately correlated with Health-ITUES scores (*r_s_*=0.43).

### Participant Feedback and Researcher Observations

#### Overview

Feedback was collected from 21 participants. Additionally, researchers provided written observations during every gameplay session. The combined feedback and observations illustrated the accessibility of the TPS, the overall experience of gameplay, and areas of future improvement.

#### Accessibility of the System

The most frequent comment about accessibility reported by our participants was that the TPS was easy and simple to use. A few participants noted that they could not play typical sedentary video games due to a lack of hand dexterity, and the TPS enabled them to participate in video games.

They (touch pads) helped me play the games rocking back and forth. Hitting the back, moving in each direction. So better than me pressing a joystick up and down.

Man, 40 years, cerebral palsy

Researchers observed and participants suggested aspects of the TPS that were barriers to accessibility such as the touchpads moving and drifting position during gameplay. Applying a sandbag weight to the touchpad stands reduced movement and drift. Because the TPS relies on skin contact to activate each touchpad, a few participants occasionally encountered difficulty activating a pad. Researchers were able to mitigate this barrier by applying a small amount of moisturizer to the participant’s skin. Amputee participants occasionally encountered difficulty activating a touchpad with their prosthetic; however, they were able to after a researcher placed a small amount of conductive tape on the surface of the prosthetic. A couple of participants who used a manual wheelchair did not have brakes, and blocks were needed behind their wheels to keep their chair from drifting.

Right now, it is a neutral (touch) pad, being a visual person...colors would help me which ones go which way.

Woman, 32 years, amputee

No difficulty activating the pads, but the stands swiveled and moved out of their way and needed repositioning.

Researcher’s note

#### Experience Using TPS for Video Game Play

Participants were asked to describe their overall experience playing video games with the TPS. Comments were mostly positive about their play experience. The most common response was that their experience was enjoyable.

I think other people will enjoy it as much as I did, especially if you are in a wheelchair.

Woman, 21 years, hydrocephalus

I think it’s going to be good especially with people with lot lesser ability.

Woman, 48 years, spina bifida

Participants described the experience as novel, intuitive, and responsive. Some participants also remarked that they were motivated to play again in the future. Two participants with hemiparesis liked that they could use their affected side during gameplay.

I like the touch pads because I was able to use my impaired limb during play.

Woman, 42 years, poststroke

One participant noted that gameplay with the TPS was not as physically demanding as they anticipated it would be. Another participant expressed concern about the time necessary to become proficient using the TPS.

It was exciting, but had never done it before…I think it would take time to master.

Man, 58 years, spinal cord injury

#### Future Iteration or Areas of Improvement

Participants provided many suggestions, and researchers observed several areas to improve the TPS for future use. Many of our participants suggested that they try sports games with the TPS. It was suggested by participants and researchers alike that the touchpads should be mounted in a way that prevents the pad from moving. Another suggestion is that we use color, letter, or other visual systems to quickly let players know which action was associated with each touchpad. It was noted that the TPS be revised so as not to require skin contact. Finally, it was suggested that the next iteration of the TPS feature a solution to address using a wheelchair without brakes.

## Discussion

### Principal Findings

We assessed the usability of the TPS and explored the enjoyment, perceived exertion, task self-efficacy, participant feedback, and researcher observations. In total, 21 individuals with impaired mobility played several video game titles (Table 1) while sitting in a chair, standing, or sitting in their own wheelchairs. A promising result was that every participant was able to use the TPS to play all video game titles for at least 5 minutes; however, numerous modifications were needed to foster the experience for many of the participants.

Consistent with other AVG controllers for people with impaired mobility, the participants found the TPS usable (Table 3) [13] However, the Health-ITUES scores were slightly below the suggested benchmark [44]. Only the participants who used the TPS standing reported a minimum score above the usability threshold of 68. The participants who played sitting in a chair reported a mean score barely above the threshold. This suggests the TPS can be improved to be more usable to seated players. Additionally, our overall Health-ITUES mean did not meet the suggested cutoff score of 4.32. Interestingly, our chair-sitting group reported the lower usability scores with higher variability than those who played sitting in their own wheelchairs, which is not consistent with the usability results of another AVG controller we were testing in our laboratory [13]. The chair we provided gameplay was consistent but every wheelchair player used their own device. Therefore, participants’ own personalized devices likely provide a more comfortable gameplay environment, which may have affected their usability scores.

Similar to previous studies using AVGs among individuals with impaired mobility, participants moderately enjoyed using the TPS (Table 4) [20] Similar to their peers without impaired mobility, participants had moderate to high self-efficacy using the TPS before and after gameplay [34,49]. Our lowest self-efficacy scores were reported by participants who played sitting in a chair, who also reported the lowest usability, enjoyment, and highest perceived exertion. It is possible that the participants sitting in a chair to use the TPS had a less positive overall experience than their peers who played standing or from their own wheelchair. The only 2 participants who reported below moderate enjoyment were individuals’ poststroke who reported not playing video games (0 minutes per week). Both individuals also reported low self-efficacy postplay and low usability scores. While AVGs can enhance self-efficacy [29,32], lower enjoyment and lack of experience playing AVGs may reduce self-efficacy.

These data suggest that our participants perceived their exertion as somewhat easy to somewhat hard during video game play using the TPS (Table 4), which is consistent among individuals with neuromuscular conditions [50]. It is unclear why the 3 participants with the highest mean perceived exertion were men >50 years. To better understand the influence of the warmup game (Flower) on perceived exertion, we calculated an exercise RPE by removing all the warmup game RPE values. This did not alter exertion. Our observed perceived exertion scores are consistent with RPE observed from previous TPS testing [34]. Our RPE findings are also comparable to scores reported by adults with impaired mobility, playing video games using an adapted Nintendo Wii balance board and an adapted gaming mat from a sitting and standing position [18,20].

The participants regarded the touchpads as novel, fun, and entertaining but they did encounter some accessibility barriers. Modifications such as repositioning the touchpads, adding conductive tape to prosthetics, and providing moisturizer for dry skin were not anticipated because they were not encountered in previous testing of the TPS [34] or with a similar controller in our laboratory [13]. While successful efforts were made during the study to overcome accessibility barriers and enable players who had difficulty using the palm of their hand to contact the touchpads, the need to adjust the position of the touchpads multiple times in a single session may have detracted from the participant’s experience. The subgroup that encountered the most frequent difficulty contacting the touchpads using their palm played sitting in a chair, which may account for the lower usability among this subgroup.

For future iterations, the stands used to mount the touchpads need to resist movement when a player exerts considerable force to activate. Due to the large body movements and quick reactions required to play video games using the TPS, the touchpads need to stay fixed in position during gameplay. Even though each touchpad was made large to mitigate the need for movement precision, touchpad movement during gameplay may cause the player to lose screen focus and thus introduce frustration. The TPS needs to remain robust to a varying degree of force from multiple directions to better accommodate individuals with impaired mobility. The TPS is currently being refined based on usability, participant feedback, and researcher observations. This system can be refined to work with adaptive video game equipment such as the Microsoft Adaptive Controller. Because the TPS is not limited to a single user, future research should examine the use of this system for multiple players simultaneously.

### Limitations

First, the participants in this sample represented various mobility impairments; therefore, the number of players within each mode of play is few, which makes mode comparisons difficult. Second, our participants reported a wide range of weekly physical activity minutes (Table 2), limiting our ability to generalize these results to sedentary individuals with impaired mobility, and it is possible that physically active individuals may find the TPS more usable. Third, video game selection was limited to specific titles we felt could be demonstrated and learned quickly. Therefore, we chose video game titles that required simple commands and may not be indicative of more complex games. Additionally, the featured video games may not have appeal to some participants. Finally, we did not standardize touchpad placement relative to the player due to varied levels of dexterity. We used a consistent touchpad layout, and modifying the touchpad layout may limit the generalizability of our results; however, we feel this decision engendered rich feedback regarding the accessibility of the TPS.

### Conclusions

The TPS enabled people with impaired mobility to participate in AVG play. The TPS allows typical sedentary video games to be played by adults with impaired mobility while sitting, standing, and with their own mobility aids. Participants found the TPS to be usable, experienced moderate enjoyment, and achieved moderate intensity physical activity through gameplay. Key areas of improvement to the system were identified based on our measures, participant feedback, and observation.

## Appendix

Multimedia Appendix 1 Video game play appraisal.

Multimedia Appendix 2 Semistructured interview guide.

## Abbreviations

AVG: active video game
Health-ITUES: Health Information Technology Usability Evaluation Scale
ICC: intraclass correlation coefficient
RPE: ratings of perceived exertion
SUS: System Usability Scale
TPS: touchpad system

## Data Availability

The data sets generated and analyzed during this study are available from the corresponding author upon reasonable request.
