# Supplementary material for: The Usability of a Touchpad Active Video Game Controller for Individuals With Impaired Mobility: Observational Study
Source: JMIR Rehabil Assist Technol. 2023 Aug 3;10:e41993. doi: 10.2196/41993 (PMC10436121; doi:10.2196/41993)
Supplement: Multimedia Appendix 1 [file rehab_v10i1e41993_app1.docx]

**Video Game Play Appraisal**

Please indicate how certain you are regarding each of the videogame play aspects listed below.

*Rate your degree of certainty by recording a number from 0 to 10 using the scale provided.*

0 1 2 3 4 5 6 7 8 9 10
No Moderate Absolute
certainty certainty certainty

**Certainty (0 to 10)**

Maintaining focus for at least 5-minutes ________________

Seeing and hearing all of the game information ________________

Reacting fast enough to choose a next action ________________

Determining strategies to move during play ________________

Coordinating body movements to carry out a strategy ________________

Moving well enough to maintain successful play ________________
