# Supplementary material for: The Usability of a Touchpad Active Video Game Controller for Individuals With Impaired Mobility: Observational Study
Source: JMIR Rehabil Assist Technol. 2023 Aug 3;10:e41993. doi: 10.2196/41993 (PMC10436121; doi:10.2196/41993)
Supplement: Multimedia Appendix 2 [file rehab_v10i1e41993_app2.docx]

**Usability Interview – Adapted Video Game Controllers**

*Thanks for participating today. We’d like to discuss certain features of each system with you. Please describe your experience in as much detail as you can.*

**Have your video gaming habits changed over time? If so, please explain?**

**Adapted Board**

Ease of mounting/dismounting the gaming board?

Did assistive device inhibit gaming activities? What if anything did you do to compensate?

Ease of playing games using the gaming board?

Ease of using accessory buttons for game play (e.g., jump, shoot, accelerate)

Use of handrails during gameplay? How did they contribute to your game play?

Did moving your trunk (leaning) provide a responsive input for game control?

What if any additional features or functions would you like to see incorporated into the gaming board?

How was your overall experience using the gaming board?

Any other comments or feedback?

**Touch Pads**

Ease of playing games using the Touch Pads?

Did the Touch Pads device inhibit your gameplay?

Were the Touch Pads responsive enough for you? Too Sensitive?

What if any additional features or functions would you like to see incorporated into the Touch Pads?

How was your overall experience using the Touch Pads?

Any other comments or feedback?
